# Supplementary material for: Is Gender an Important Factor in the Precision Medicine Approach to Levocetirizine?
Source: Pharmaceutics. 2024 Jan 21;16(1):146. doi: 10.3390/pharmaceutics16010146 (PMC10818372; doi:10.3390/pharmaceutics16010146)
Supplement: Supplementary file 1 [file pharmaceutics-16-00146-s001.zip › pharmaceutics-2817683-supplementary.pdf]

pharmaceutics

Supplementary Materials: Is Gender an Important Factor in the Precision Medicine Approach to Levocetirizine?

Seung-Hyun Jeong, Ji-Hun Jang and Yong-Bok Lee

1. Determination of Biochemical Parameters

Blank plasma samples obtained immediately before levocetirizine administration (as 0 h) were used for the analysis of clinical biochemical parameters. The types of biochemical parameters included total proteins, albumin, globulin, alkaline phosphatase (ALP), aspartate transaminase (AST), alanine transaminase (ALT), cholesterol, total bilirubin, glucose, blood urea nitrogen, gamma-glutamyl transferase (GGT), creatinine, creatinine clearance (CrCL), and estimated glomerular filtration rate (eGFR). Hematological tests included white blood cell, red blood cell, platelet, neutrophil, lymphocyte, and eosinophil counts, and hemoglobin and hematocrit. Determination of clinical biochemical parameter values was performed by serological analysis through a dry automated analyzer. The analytical instrument was the VITROS MicroSlides (Ortho Clinical Diagnostics, Raritan, NJ, USA) operated by reflectance spectrophotometry. The CrCL was calculated based on the Cockcroft-Gault equation (taking into account the gender of each individual), which was: [(140 - age) × body weight (kg) × 0.85 (if female) or 1 (if male)/(serum creatinine [mg/dL] × 72)]. eGFR was calculated using the modification of diet in renal disease (MDRD) formula (taking into account the gender of each individual), which was: 175 × [serum creatinine (mg/dL)$^{-1.154}$ × age (year)$^{-0.203}$ × 0.742 (if female) or 1 (if male)]. Body mass index (BMI) and body surface area (BSA) were calculated based on the measurement information about the weight and height of the individuals. The BMI was calculated based on the Kaup index, which was: [body weight (kg)/height^2 (m^2)]. The BSA was calculated based on the Mosteller formula, which was: $\sqrt{(\text{height [cm]} \times \text{ weight [kg]}/3600)}$. Focusing on the possibility of variations in the pharmacokinetics of levocetirizine depending on renal function, additional calculations of the related indicators (CrCL based on ideal body weight [IBW], CrCL according to BMI-based weight selection, chronic kidney disease epidemiology collaboration [CKD-EPI] GFR, and CKD-EPI GFR adjusted to BSA) were performed. CrCL based on the IBW was calculated by applying the IBW instead of the body weight in the CrCL calculation formula (Cockcroft-Gault equation), and the IBW was calculated by adding 2.3 kg for each inch over 5 feet from 45.5 kg (if female) or 50 kg (if male). The CrCL according to the BMI-based weight selection calculates the CrCL while considering the BMI of each individual. In the existing CrCL formula (Cockcroft-Gault equation), if the BMI is more than 23 kg/m^2, the IBW is reflected, and if the BMI is less than 23 kg/m^2, the actual measured body weight is reflected. The CKD-EPI GFR has even been reported to more accurately predict the GFR than the MDRD formula in various populations, including kidney transplant patients, which was calculated as 141 × [serum creatinine (mg/dL)/0.7 (if female) or 0.9 (if male)$^{-0.329\text{ (if male) or }-0.411\text{ (if male)}}$] × [serum creatinine (mg/dL)/0.7 (if female) or 0.9 (if male)$^{-1.209}$] × 0.993$^{\text{age (year)}}$ × 1.018 (if female) or 1 (if male)]. The CKD-EPI GFR adjusted to the BSA was calculated by multiplying the estimated CKD-EPI GFR considering the BSA in the GFR by BSA/1.73 m^2 for each individual. This was an attempt to additionally estimate the overall eGFRs while considering the BSA in each individual, as the CKD-EPI GFR is normalized to 1.73 m^2.

2. Subjects

The number of subjects recruited to conduct this clinical trial was 68, of which 40 who met the selection criteria without meeting the exclusion criteria participated in the final test. All 40 people who participated in the trial underwent thorough health examinations (vital signs, physical examination, electrocardiogram, hematology, blood chemistry,

urine, and serology tests, etc.), no clinically significant adverse reactions such as abnormal values or findings were identified, and no concomitant medications were administered. All subjects provided written informed consent before participating in the clinical trial. This clinical study was conducted in accordance with the Declaration of Helsinki as implemented in the Good Clinical Practice guidelines. The clinical trial was conducted at the Bumin Clinical Trial Center (Seoul, Republic of Korea) and the total duration was from May 25, 2022 to June 12, 2022. This included the follow-up observation period of the subjects from the start of administration of the test drug to the end of administration.

3. Clinical Trial Design and Sampling

This bioequivalence study was performed as randomized, single-dose, open-label, crossover, and two-way studies (with a washout period of 7 days). Prior to the clinical trial, subjects had a heparin-locked (150 unit/mL) JELCO 22G angio-catheter (Smiths Medical, Minneapolis, MN, USA) installed in a vein on the arm or back of the hand, and 6 mL of blank blood was collected. All subjects were then given a single dose of levocetirizine tablet 5 mg orally with 150 mL of water. Subjects were fasted for more than 10 h before administration. Blood sampling from subjects was performed at a total of 15 points after administration and the specific time points were as follows: Before administration (0 h) and at 0.17, 0.33, 0.5, 0.75, 1, 1.25, 1.5, 2, 3, 4, 6, 8, 12, 24, and 36 h after oral administration. To completely remove the heparinized saline solution remaining in the venous catheter during blood collection, approximately 1 mL of blood was discarded each time, and approximately 6 mL of blood was collected and placed in sodium heparinized Vacutainer tubes (BD, Franklin Lakes, NJ, USA) labeled with the subject management number and blood collection time. After each blood sampling (draw), saline containing heparin was administered to prevent clotting of the blood remaining in the intravenous catheter. The collected blood was centrifuged at 3,000 × *g* for 10 min in a centrifuge set at 4°C, and then about 1 mL of plasma was taken and transferred to an Eppendorf tube (Eppendorf, Hamburg, Germany) and stored at −80°C until analysis.

4. Determination of Levocetirizine Plasma Concentrations

Concentrations of unchanged levocetirizine in plasma were determined by applying ultrahigh performance liquid chromatography coupled with a mass spectrometer, which was established and validated in previous studies. A Waters ACQUITY UPLC/Xevo® TQ-XS (Waters Corp., Milford, MA, USA) was used as the analytical instrument. To briefly describe the analysis method, plasma samples were pretreated through protein precipitation using acetonitrile, and then levocetirizine was selectively separated on an Imtakt Unison UK-C_{18} (3 μm, 2.0 × 100 mm) column using 0.1% (v/v) formic acid in 10 mM ammonium formate (mobile phase A) and acetonitrile (mobile phase B) as the mobile phases. The composition ratio of mobile phases A and B was 50:50 (v/v) and isocratic elution was performed; the run time per sample was as short as 3 min. Quantification of levocetirizine was performed by positive electrospray ionization using the multiple reaction monitoring mode. Levocetirizine-d_4 was used as an internal standard (IS), and the mass transitions of levocetirizine and IS were 389.10 → 201.00 and 393.00 → 200.90, respectively. The retention times in the column for levocetirizine and IS were the same at 0.98 min. The lower limit of quantification was 0.5 ng/mL, and the linear calibration curve was secured with a coefficient of determination of 0.99 or higher up to the concentration range of 500 ng/mL. The concentration values of all plasma samples did not exceed the upper limit of the calibration curve (500 ng/mL). The intra- and inter-day precision and accuracy of the method were all within a 15% coefficient of variation (CV), and no significant carryover or matrix effect was identified. Additionally, the stabilities of levocetirizine and IS in the plasma samples were all above 90%, showing no problems.

5. Non-Compartment Analysis

Calculations of the basic pharmacokinetic parameters of levocetirizine were performed by non-compartment analysis (NCA). Phoenix WinNonlin software (version 8.4, Certara Inc., Princeton, NJ, USA) was used for pharmacokinetic parameter estimation. The area under the curve from 0 h to infinite (AUC_{inf}) was calculated as the sum of the AUC_{all} and C_{last}/k, where the C_{last} is the final measured concentration, t is the time in C_{last}, and k is the elimination rate constant at the terminal phase. The AUC_{all} was calculated using a linear trapezoidal rule from 0 to t h after oral administration of the levocetirizine 5 mg tablet. The area under the first-order moment curve (AUMC) was calculated as the area of the graph under the product of time and the levocetirizine plasma concentration over time. The mean residence time (MRT) was obtained as the ratio of the AUMC and AUC. The half-life ($T_{1/2}$) was calculated as 0.693/k, and the volume of the distribution (V/F) was calculated as dose/k · AUC_{inf}. The clearance (CL/F) was calculated by dividing the levocetirizine dose by the AUC_{inf}, where F is the bioavailability of oral administration. The peak plasma drug concentration (C_{max}) and time to reach C_{max} (T_{max}) were determined from the levocetirizine plasma concentration-time curves of each individual after oral administration of the levocetirizine 5 mg tablet.

6. Model Qualification Tools

The goodness of fit (GOF) was confirmed by using diagnostic scatter plots as follows: conditional weighted residuals (CWRES) versus time after dose (IVAR); observed concentrations (DV) versus individual predicted concentrations (IPRED); DV versus population predicted concentrations (PRED); quantile-quantile (QQ) plot of the components of CWRES; CWRES versus PRED; and relative frequency of CWRES. The visual predictive check (VPC) of the final established model was performed by using the VPC option of Phoenix NLME (version 8.4, Certara Inc., Princeton, NJ, USA). Additionally, the replicative number of simulations for the VPC was 1,000. The DV-IVAR concentration data were graphically superimposed on the median values and the 2.5^{th} and 97.5^{th} percentiles of the simulated concentration-IVAR profiles. If the DV data were approximately distributed within the 2.5^{th} and 97.5^{th} prediction interval (containing 95% confidence intervals for each), the model was considered precise and had no major problems with its outcome predictions. By using non-parametric bootstrap analysis, the stability and robustness of the final model were confirmed, which was conducted by using the bootstrap option of Phoenix NLME. A total of 1,000 replicates were generated by repeated random sampling with replacement from the original dataset. The estimated parameter values, such as the confidence interval and medians from the bootstrap procedure, were compared with those (such as the typical value estimates and standard error) estimated from the original dataset.

7. Population Pharmacokinetic Model Equations For The Levocetirizine Tablet

The formula for the final established population pharmacokinetic model (based on the results of the pharmacokinetic profile obtained after single oral administration of the levocetirizine 5 mg tablet) parameters of levocetirizine was as follows:

$$K_{a1} = tv\ K_{a1} \cdot \exp(\eta_{Ka1}) \quad (1)$$

Copyright: © 2024 by the authors. Licensee MDPI, Basel, Switzerland. This article is an open access article distributed under the terms and conditions of the Creative Commons Attribution (CC BY) license (https://creativecommons.org/licenses/by/4.0/).

$$K_{a2} = tv\ K_{a2} \cdot \exp(\eta_{Ka2}) \quad (2)$$

$$K_{a3} = tv\ K_{a3} \cdot \exp(\eta_{Ka3}) \quad (3)$$

$$K_{a4} = tv\ K_{a4} \cdot \exp(\eta_{Ka4}) \quad (4)$$

$$K_{a5} = tv\ K_{a5} \cdot (\text{Weight}/\text{median Weight})\text{\^{}}dK_{a5}d\text{Weight} \cdot \exp(\eta_{Ka5}) \quad (5)$$

$$\frac{Vc}{F} = tv\ \frac{Vc}{F} \cdot \exp(\eta_{Vc/F}) \quad (6)$$

$$\frac{CLc}{F} = tv\ \frac{CLc}{F} \cdot \exp(\eta_{CLc/F}) \quad (7)$$

$$T_{lag} = tv\ T_{lag} \cdot \exp(\eta_{Tlag}) \quad (8)$$

$$\frac{Vp}{F} = tv\ \frac{Vp}{F} \cdot (\text{BSA}/\text{median BSA})\text{\^{}}dV_p/Fd\text{BSA} \cdot (\text{Albumin}/\text{median Albumin})\text{\^{}}dV_p/Fd\text{Albumin} \cdot [1 + dV_p/Fd\text{Gender} \cdot (\text{If Male} = 0 \text{ or Female} = 1)] \cdot \exp(\eta_{Vp/F}) \quad (9)$$

$$\frac{CLp}{F} = tv\ \frac{CLp}{F} \cdot (\text{BSA}/\text{median BSA})\text{\^{}}dCL_p/Fd\text{BSA} \cdot \exp(\eta_{CLp/F}) \quad (10)$$

where K_{a1} is the first sequential absorption (dosing depot-to-depot 1) rate constant, K_{a2} is the second sequential absorption (depot 1-to-depot 2) rate constant, K_{a3} is the third sequential absorption (depot 2-to-depot 3) rate constant, K_{a4} is the fourth sequential absorption (depot 3-to-depot 4) rate constant, K_{a5} is the fifth sequential absorption (depot 4-to-central compartment) rate constant, V_c/F is the central compartment distribution volume, CL_c/F is the central compartment clearance, T_{lag} is the lag-time, V_p/F is the peripheral compartment distribution volume, and CL_p/F is the peripheral compartment clearance. The tv means typical parameter values. The median weight, median BSA, and median albumin represent the median body weight, body surface area (BSA), and albumin values in the observed population, respectively. The $dK_{a5}d$Weight means the degree of correlation between K_{a5} and body weight. The dV_p/FdBSA means the degree of correlation between the V_p/F and BSA. The dV_p/FdAlbumin means the degree of correlation between the V_p/F and albumin. The dV_p/FdGender means the degree of correlation between the V_p/F and gender. The dCL_p/FdBSA means the degree of correlation between the CL_p/F and BSA. The inter-individual variabilities in the pharmacokinetic parameters of levocetirizine were evaluated by using an exponential error model, as shown in the following equation: $P_i = P_{tv} \cdot \exp(\eta_i)$, where η_i is the random variable for the ith individual, which was normally distributed with mean 0 and variance ω^2, P_i is the parameter value of the ith individual, and P_{tv} is the typical value of the population parameter.

8. Pharmacodynamic Model Equation For The Levocetirizine Tablet

The formula for the final established pharmacodynamic model (based on the results of the pharmacokinetic and pharmacodynamic profiles obtained after single oral administration of the levocetirizine 5 mg tablet) of levocetirizine was as follows:

$$\frac{dR}{dt} = K_{in} \times \left(1 - \frac{I_{max} \times C^{\gamma}}{IC_{50}^{\gamma} + C^{\gamma}}\right) - K_{out} \times R \quad (11)$$

where R stands for the antihistamine effect (as percent inhibition of wheal or flare size induced by exogenous histamine) induced by levocetirizine. dR/dt refers to the percent change in the antihistamine effect over time after exposure to levocetirizine. K_{in} means the zero-order rate constant for increasing the percent change in the wheal area or flare relative to the basal value. K_{out} means the first-order rate constant for decreasing the percent change in the wheal area or flare relative to the basal value. I_{max} means the maximum fractional extent of inhibition. IC_{50} means the plasma concentration of levocetirizine that inhibits K_{in} by 50%. C means the concentration of levocetirizine in the plasma. γ means the shape function of the profile.

pharmaceutics MDPI

Table S1. Demographic information of healthy subjects participating in a single oral exposure clinical trial of levocetirizine 5 mg tablet.

Parameters	Total (n = 40)	Male (n = 24)	Female (n = 16)	p value (* means < 0.05)
Age (year)	28.38 ± 8.30	28.58 ± 8.08	28.06 ± 8.88	0.85
Height (cm)	168.33 ± 9.10	173.41 ± 7.50	160.70 ± 5.08	$7.23 \times 10^{-7*}$
Weight (kg)	66.97 ± 12.59	71.58 ± 12.81	60.05 ± 8.68	$3.24 \times 10^{-3*}$
Ideal body weight (kg)	62.69 ± 9.96	69.12 ± 6.82	53.05 ± 4.62	$5.72 \times 10^{-10*}$
Body surface area (m^2)	1.76 ± 0.20	1.85 ± 0.19	1.63 ± 0.13	$3.11 \times 10^{-4*}$
Body mass index (kg/m^2)	23.53 ± 3.36	23.73 ± 3.59	23.24 ± 3.06	0.66
Blood urea nitrogen (mg/dL)	11.99 ± 2.71	12.71 ± 2.79	10.91 ± 2.26	0.04
Serum creatinine (mg/dL)	0.79 ± 0.16	0.90 ± 0.11	0.62 ± 0.04	$3.78 \times 10^{-13*}$
Estimated glomerular filtration rate (mL/min/1.73 m^2)	108.25 ± 15.85	102.78 ± 16.84	116.44 ± 9.92	$2.60 \times 10^{-3*}$
Total protein (g/dL)	7.47 ± 0.36	7.51 ± 0.40	7.40 ± 0.30	0.62
Albumin (g/dL)	4.70 ± 0.24	4.79 ± 0.23	4.57 ± 0.17	$2.75 \times 10^{-3*}$

Alkaline phosphatase (U/L)	71.48 ± 16.70	78.33 ± 15.40	61.19 ± 13.16	$7.88 \times 10^{-4*}$
Aspartate transaminase (U/L)	24.78 ± 4.73	25.08 ± 4.83	24.31 ± 4.70	0.62
Alanine transaminase (U/L)	26.05 ± 15.05	27.58 ± 17.66	23.75 ± 10.07	0.39
Gamma-glutamyl transferase (U/L)	26.08 ± 21.93	32.25 ± 25.96	16.81 ± 8.06	$1.08 \times 10^{-2*}$
Total bilirubin (mg/dL)	0.93 ± 0.32	1.00 ± 0.36	0.83 ± 0.21	0.06
Total cholesterol (mg/dL)	187.73 ± 30.92	182.17 ± 30.22	196.06 ± 31.00	0.17
Creatinine clearance based on weight (mL/min)	125.91 ± 23.70	124.05 ± 24.34	128.70 ± 23.18	0.55
Creatinine clearance based on ideal body weight (mL/min)	117.97 ± 19.73	120.76 ± 21.93	113.79 ± 15.63	0.28
Creatinine clearance according to body mass index-based weight selection (mL/min) [a]	114.84 ± 18.88	114.53 ± 20.68	115.31 ± 16.44	0.90

CKD-EPI glomerular filtration rate (mL/min/1.73 m²)	129.08 ± 25.56	119.30 ± 26.56	143.74 ± 15.31	7.36×10^{-4}*
CKD-EPI glomerular filtration rate adjusted to body surface area (mL/min)	130.50 ± 24.21	126.99 ± 27.16	135.76 ± 18.55	0.27

CKD-EPI: chronic kidney disease-epidemiology collaboration equation. Parameter values are presented as mean ± standard deviation, respectively. [a] Individuals with a body mass index value of 23 kg/m² or higher were classified as overweight and the ideal body weight value was applied, while those with a body mass index value of less than 23 kg/m² were classified as normal weight and the actual body weight value was applied. *$p < 0.05$ compared between males and females.

Table S2. Non-compartmental analysis results calculated based on plasma concentration values following single oral exposure of the levocetirizine 5 mg tablet.

Parameters	Total (n = 40)	Male (n = 24)	Female (n = 16)	p value (* means < 0.05)
AUC_{all} (h·ng/mL)	1702.05 ± 304.69	1647.94 ± 316.34	1783.22 ± 276.08	0.17
AUC_{inf} (h·ng/mL)	1784.67 ± 353.54	1751.08 ± 387.40	1835.06 ± 300.56	0.47
AUC_{extrap} (%)	4.33 ± 2.62	5.42 ± 2.74	2.69 ± 1.27	NA
C_{max} (ng/mL)	214.67 ± 37.22	203.89 ± 30.37	230.84 ± 41.51	0.02*
T_{max} (h)	1.11 ± 0.72	1.11 ± 0.83	1.09 ± 0.55	0.92
$T_{1/2}$ (h)	8.06 ± 1.56	8.73 ± 1.53	7.05 ± 0.96	1.23×10^{-4}*
CL/F (mL/h)	2900.98 ± 531.04	2973.71 ± 577.87	2791.89 ± 447.23	0.29
V/F (mL)	33135.56 ± 6142.21	36538.29 ± 5051.72	28031.46 ± 3555.93	9.73×10^{-7}*
MRT (h)	10.81 ± 2.24	11.79 ± 2.24	9.34 ± 1.23	7.24×10^{-5}*

AUC_{all}: area under the curve from 0 to observed (t) time after administration; AUC_{inf}: area under the curve from 0 to infinity time after administration; AUC_{extrap}: fraction of AUC_{inf} and the area under the curve from the observed (t) time to infinity after administration; C_{max}: maximum plasma concentration; T_{max}: time to reach C_{max}; $T_{1/2}$: half-life; CL: clearance; F: oral bioavailability; V: volume of distribution; MRT: mean residence time; NA: not applicable (because the results are meaningless). *$p < 0.05$ compared between males and females. Parameter values are presented as mean ± standard deviation, respectively.

Table S3. Non-compartmental analysis results calculated based on values of plasma concentrations normalized to the body weight following single oral exposure to the levocetirizine 5 mg tablet.

Parameters	Total (n = 40)	Male (n = 24)	Female (n = 16)	p value (* means < 0.05)
AUC_{all} (h·ng/mL/kg)	26.57 ± 7.93	23.90 ± 6.89	30.58 ± 7.89	7.29×10^{-3}*
AUC_{inf} (h·ng/mL/kg)	27.80 ± 8.39	25.36 ± 7.68	31.47 ± 8.28	0.02*
C_{max} (ng/mL/kg)	3.36 ± 1.01	2.97 ± 0.84	3.94 ± 0.98	1.93×10^{-3}*
WNCL/F (mL· kg/h)	196114.23 ± 58126.74	213911.66 ± 60171.15	169418.08 ± 44341.14	0.02*
WNV/F (mL·kg)	2270558.03 ± 782624.31	2649393.17 ± 744069.96	1702305.31 ± 411731.76	8.54×10^{-6}*

AUC_{all}: area under the curve from 0 to observed (t) time after administration; AUC_{inf}: area under the curve from 0 to infinity time after administration; C_{max}: maximum plasma concentration; WNCL: estimated clearance based on plasma levocetirizine concentrations normalized to body weight; F: oral bioavailability; WNV: estimated volume of distribution based on plasma levocetirizine concentrations normalized to body weight. *$p < 0.05$ compared between males and females. Parameter values are presented as mean ± standard deviation, respectively.

Table S4. Non-compartmental analysis results calculated based on values of plasma concentrations normalized to the body surface area following single oral exposure to the levocetirizine 5 mg tablet.

Parameters	Total (n = 40)	Male (n = 24)	Female (n = 16)	p value (* means < 0.05)
AUC_{all} (h·ng/mL/m^2)	983.72 ± 239.92	903.30 ± 214.62	1104.35 ± 230.56	7.63×10^{-3}*

AUC_{inf} (h·ng/mL/m²)	1030.10 ± 259.01	959.22 ± 248.21	1136.42 ± 244.68	0.03*
C_{max} (ng/mL/m²)	124.20 ± 30.40	112.05 ± 24.64	142.44 ± 29.67	1.14×10^{-3}*
BNCL/F (mL· m²/h)	5150.77 ± 1257.84	5525.93 ± 1307.87	4588.03 ± 963.27	0.02*
BNV/F (mL·m²)	59352.98 ± 16745.93	68203.85 ± 14961.42	46076.68 ± 8516.40	7.49×10^{-7}*

AUC_{all}: area under the curve from 0 to observed (t) time after administration; AUC_{inf}: area under the curve from 0 to infinity time after administration; C_{max}: maximum plasma concentration; BNCL: estimated clearance based on plasma levocetirizine concentrations normalized to body surface area; F: oral bioavailability; BNV: estimated volume of distribution based on plasma levocetirizine concentrations normalized to body surface area. *$p < 0.05$ compared between males and females. Parameter values are presented as mean ± standard deviation, respectively.

Table S5. Summary of building step procedure results for establishing a basic pharmacokinetic structural model of levocetirizine.

Model	Description	nParameter [a]	Twice the negative log likelihood (−2LL)	Akaike's information criterion (AIC)	△−2LL [b]	△AIC [c]	Compared model
			**Compartment disposition model**				
01	One-compartment	7	5423.53	5437.53	NA	NA	NA
02*	Two-compartments	11	4835.15	4857.15	−588.38	−580.38	01
03	Three-compartments	15	4834.41	4864.41	−0.74	7.26	02
			**Absorption model**				
02	No lag-time (T_{lag}) with first order	11	4835.15	4857.15	NA	NA	NA
02-01	Add T_{lag} with first order	13	3012.53	3038.53	−1822.62	−1818.62	02
02-02	Add T_{lag} with sequential two absorption compartment**	15	2011.82	2041.82	−1000.71	−996.71	02-01
02-03	Add T_{lag} with sequential three absorption compartment**	17	1394.24	1428.24	−1618.29	−1610.29	02-01

02-04	Add T_{lag} with sequential four absorption compartment**	19	853.41	891.41	−2159.12	−2147.12	02-01
02-05*	Add T_{lag} with sequential five absorption compartment**	21	795.05	837.05	−2217.48	−2201.48	02-01
02-06	Add T_{lag} with sequential six absorption compartment**	23	1024.69	1070.69	229.64	233.64	02-05
02-07	Add T_{lag} with non-sequential two absorption compartment**	17	2584.30	2618.30	−428.23	−420.23	02-01
02-08	Add T_{lag} with non-sequential three absorption compartment**	19	2385.77	2423.77	−626.76	−614.76	02-01
02-09	Weibull	13	3211.26	3237.26	−1623.89	−1619.89	02
02-10	Weibull two function	19	3010.85	3048.85	−1824.30	−1808.30	02
02-11	Saturation (Michaelis-Menten kinetic type)	13	3112.96	3138.96	−1722.19	−1718.19	02
02-12	Zero order	11	4962.83	4984.83	127.68	127.68	02
02-13	Mean transit time (MTT)**	15	2452.64	2482.64	−2382.51	−2374.51	02
02-14	Add T_{lag} with MTT**	17	1952.70	1986.70	−1059.83	−1051.83	02-01
**Residual error model**							
02-05	Proportional	21	795.05	837.05	NA	NA	NA
02-05-01	Additive	21	986.39	1028.39	191.34	191.34	02-05
02-05-02*	Log additive	21	−194.37	−152.37	−989.42	−989.42	02-05
02-05-03	Mixed	22	884.58	928.58	89.53	91.53	02-05
02-05-04	Power	21	953.44	995.44	158.39	158.39	02-05
**Inter-individual variability (IIV) model**							
02-05-02-01	Remove IIV of central compartment distribution volume (V_c/F)	20	-184.28	-144.28	10.09	8.09	02-05-02
02-05-02-02	Remove IIV of central compartment clearance (CL_c/F)	20	216.40	256.40	410.77	408.77	02-05-02

02-05-02-03	Remove IIV of peripheral compartment distribution volume (V_P/F)	20	318.53	358.53	512.90	510.90	02-05-02
02-05-02-04	Remove IIV of peripheral compartment clearance (CL_P/F)	20	225.68	265.68	420.05	418.05	02-05-02
02-05-02-05	Remove IIV of first absorption rate constant (K_{a1})	20	-186.87	-146.87	7.50	5.50	02-05-02
02-05-02-06	Remove IIV of second absorption rate constant (K_{a2})	20	-186.03	-146.03	8.34	6.34	02-05-02
02-05-02-07	Remove IIV of third absorption rate constant (K_{a3})	20	-185.04	-145.04	9.33	7.33	02-05-02
02-05-02-08	Remove IIV of fourth absorption rate constant (K_{a4})	20	-184.13	-144.13	10.24	8.24	02-05-02
02-05-02-09	Remove IIV of fifth absorption rate constant (K_{a5})	20	-12.52	27.48	181.85	179.85	02-05-02
02-05-02-10	Remove IIV of T_{lag}	20	48.57	88.57	242.94	240.94	02-05-02

NA: not applicable. [a] nParameter means the total number of parameters applied to the model. [b] The change value of twice the negative log likelihood according to the comparison between models. [c] The change value of Akaike's information criterion according to the comparison between models. * The model selected in each step. ** A kind of multiple absorption compartment model.

Table S6. Summary of the stepwise selection results of potential covariates (considered as preferential reflection) in the population pharmacokinetic model of levocetirizine.

Model	Description	Objective function value (OFV)	△OFV [a]	Compared with	nParameter [b]
1	Base model [c]	−194.37	NA	NA	15
2	Body surface area (BSA) on peripheral compartment distribution volume (V_P/F)	−236.91	−42.54	Base model	22
3	BSA on V_P/F and Gender on V_P/F	−258.89	−21.98	Model 2	23
4	BSA on V_P/F and peripheral compartment clearance (CL_P/F), and Gender on V_P/F	−273.83	−14.94	Model 3	24

5	BSA on V_P/F and CL_P/F, Gender on V_P/F, and Body weight on fifth absorption rate constant (K_{a5})	−282.68	−8.85	Model 4	25
6*	BSA on V_P/F and CL_P/F, Gender on V_P/F, Body weight on K_{a5}, and Albumin on V_P/F	−289.59	−6.92	Model 5	26

NA: not applicable. [a] The change of objective function value according to the comparison between models. [b] nParameter is the total number of parameters applied to the model. [c] Base model: two-compartment disposition, first-order sequential fifth absorption compartments with lag-time, log additive residual error, full consideration of inter-individual variability in parameters. * The finally selected model (through the forward selection of a 0.05 *p* value and backward elimination of a 0.01 *p* value).

Table S7. Parameter values and bootstrap results of the finally established population pharmacokinetic model for levocetirizine.

Parameter	Final model			Bootstrap (*n* = 1,000)	
	Estimate	Standard error	Coefficient of variation (%)	Median	95% confidence interval
tv V_c/F (L) [a]	1.07	0.28	26.00	1.03	0.63-1.44
tv CL_c/F (L/h) [a]	2.94	0.07	2.49	2.95	2.79-3.07
tv T_{lag} (h) [a]	0.08	0.01	10.15	0.08	0.07-0.10
tv V_P/F (L) [a]	28.12	1.07	3.82	28.02	26.44-30.70
tv CL_P/F (L/h) [a]	13.80	2.24	16.21	13.88	9.57-18.16
tv K_{a1} (1/h) [a]	9.87	1.53	15.53	9.86	7.07-12.74
tv K_{a2} (1/h) [a]	28.41	5.39	18.96	28.60	20.87-40.82
tv K_{a3} (1/h) [a]	11.53	1.87	16.20	11.45	7.91-15.35
tv K_{a4} (1/h) [a]	11.29	1.95	17.26	10.71	8.29-15.67
tv K_{a5} (1/h) [a]	0.90	0.15	16.85	0.90	0.62-1.18
dV_P/FdGender [b]	−0.18	0.02	11.35	−0.18	−0.24-−0.15
dK_{a5}dWeight [c]	0.69	0.28	40.62	0.56	0.51-1.41
dV_P/FdBSA [d]	1.20	0.14	11.59	1.18	0.94-1.54
dCL_P/FdBSA [e]	1.94	0.49	25.29	1.80	1.21-3.29
dV_P/FdAlbumin [f]	−0.79	0.19	23.90	−0.79	−1.23-−0.40
ε	0.11	0.01	11.04	0.11	0.09-0.13
$\omega^2_{Vc/F}$	0.89	0.29	33.13	0.87	0.05-1.70
$\omega^2_{CLc/F}$	0.03	0.01	23.45	0.03	0.02-0.05
ω^2_{Tlag}	0.37	0.11	29.46	0.37	0.10-0.65
$\omega^2_{Vp/F}$	0.00	0.00	33.29	0.00	0.00-0.00
$\omega^2_{CLp/F}$	0.00	0.00	0.11	0.00	0.00-0.00
ω^2_{Ka1}	2.09	0.29	13.72	2.06	1.33-2.79

ω^2_{Ka2}	4.14	0.98	23.65	3.94	2.08-5.80
ω^2_{Ka3}	2.36	0.43	18.05	2.31	1.49-3.12
ω^2_{Ka4}	2.11	0.41	19.54	2.13	1.20-3.07
ω^2_{Ka5}	0.02	0.01	34.76	0.02	0.01-0.03

V_c/F, central compartment distribution volume; CL_c/F, central compartment clearance; T_{lag}, lag-time; V_p/F, peripheral compartment distribution volume; CL_p/F, peripheral compartment clearance; K_{a1}, first sequential absorption rate constant; K_{a2}, second sequential absorption rate constant; K_{a3}, third sequential absorption rate constant; K_{a4}, fourth sequential absorption rate constant; K_{a5}, fifth sequential absorption rate constant; BSA, body surface area. [a] tv, typical value. [b] The quantitative correlation value between V_p/F and gender (as a valid categorical covariate). [c] The quantitative correlation value between K_{a4} and body weight (as a valid continuous covariate). [d] The quantitative correlation value between V_p/F and BSA (as a valid continuous covariate). [e] The quantitative correlation value between CL_p/F and BSA (as a valid continuous covariate). [f] The quantitative correlation value between V_p/F and albumin (as a valid continuous covariate).

Table S8. Typical values of levocetirizine pharmacodynamic model parameters.

Parameter	Value for wheal response	Value for flare response
K_{in} (%/h)	87.9	99.9
K_{out} (1/h)	0.879	0.999
I_{max}	0.92	0.999
IC_{50} (ng/mL)	45.5	10.5
γ	1.11	2.51

K_{in}, the zero-order rate constant for increasing the percent change in the wheal area or flare relative to the basal value. K_{out}, the first-order rate constant for decreasing the percent change in the wheal area or flare relative to the basal value. I_{max}, the maximum fractional extent of inhibition. IC_{50}, the plasma concentration of levocetirizine that inhibits K_{in} by 50%.

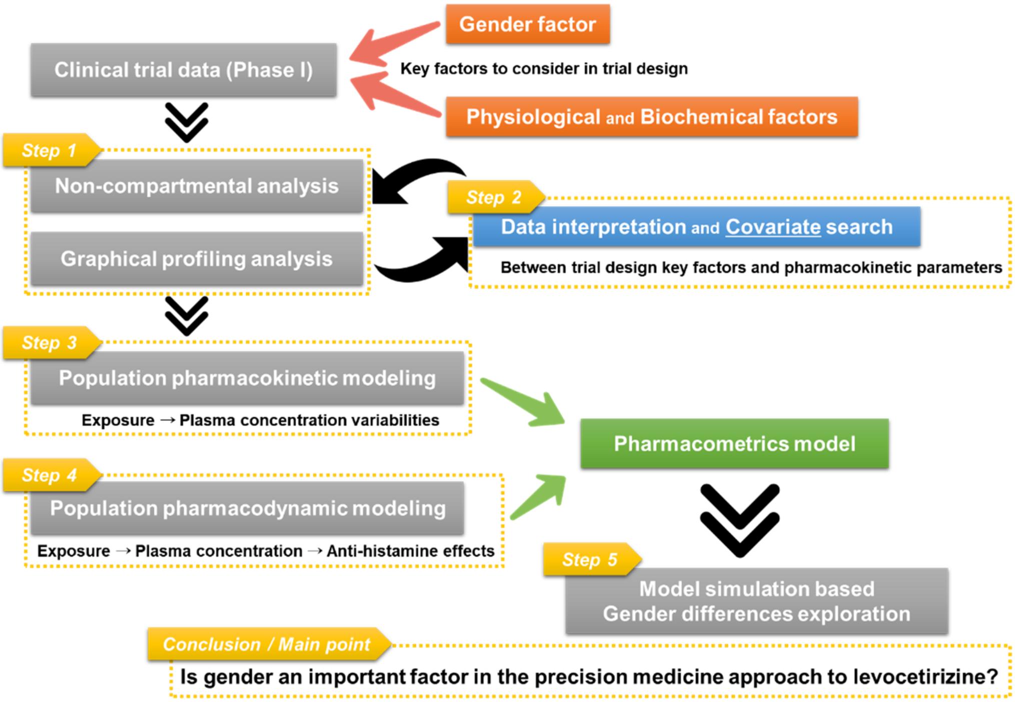

Figure S1. Schematic diagram of the approach of this study.

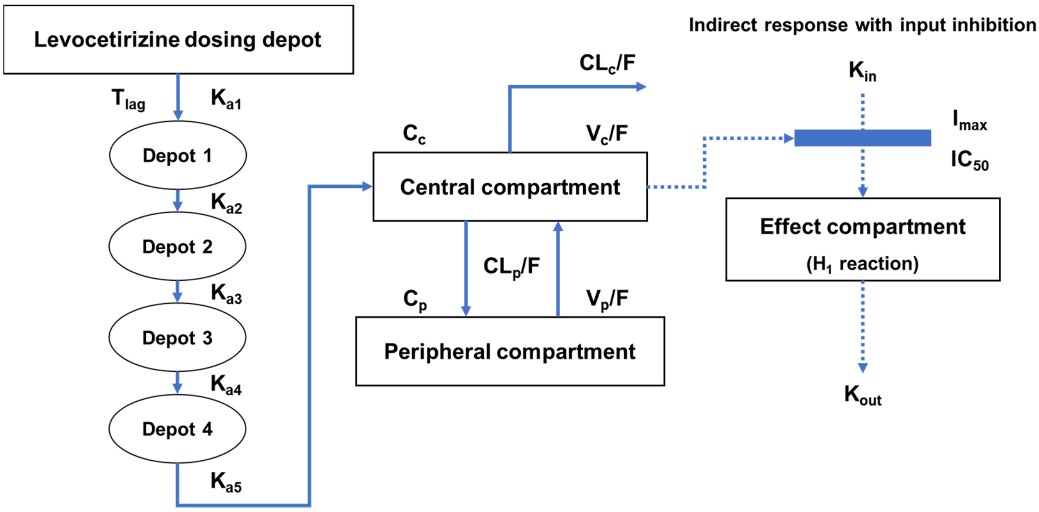

Figure S2. Structure of a population pharmacokinetic model of levocetirizine and extended linkage to a pharmacodynamic model. In the figure, K_{a1}, K_{a2}, K_{a3}, K_{a4}, and K_{a5} represent the rate constants between depots for multiple oral absorption of levocetirizine. C_c and C_p mean the concentration of levocetirizine in the central and peripheral compartments, respectively. V_c/F and V_p/F mean the distribution volume of levocetirizine in the central and peripheral compartments, respectively. CL_c/F denotes the rate of levocetirizine elimination from the central compartment and CL_p/F denotes the rate of levocetirizine movement between the central and peripheral compartments. K_{in} and K_{out} mean the zero- and first-order rate constants for increasing and decreasing the percent change in the wheal area or flare relative to the basal value, respectively. I_{max} means the maximum fractional extent of inhibition. IC_{50} means the plasma concentration of levocetirizine that inhibits K_{in} by 50%. The effect compartment was set up as an element to simulate the histamine (H_1) response inhibition percent by levocetirizine.

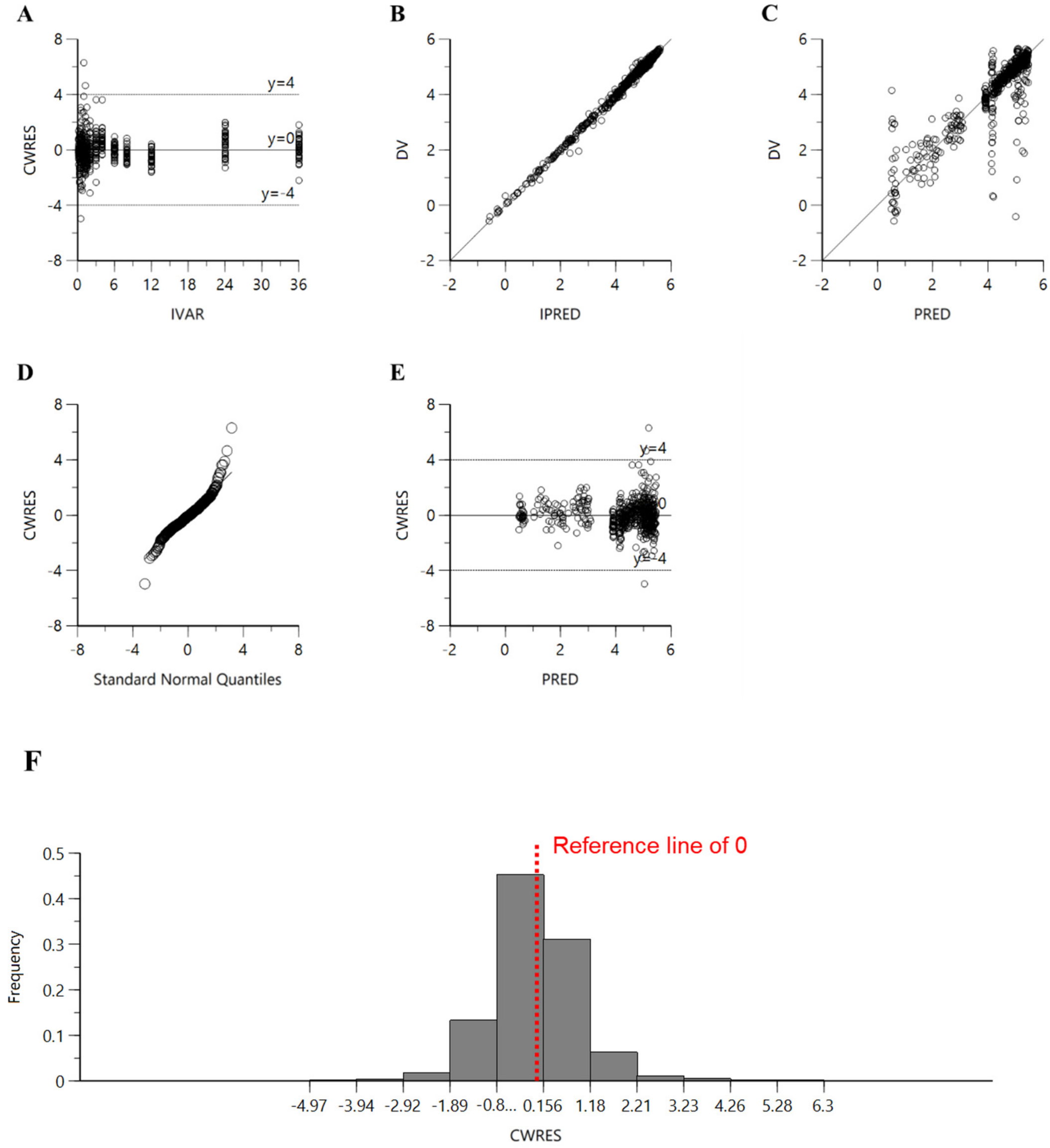

Figure S3. Goodness of fit plots of the final pharmacokinetic model for levocetirizine. (A) Conditional weighted residuals (CWRES) against time (IVAR, h); (B) Observed plasma concentration (DV, natural log scale, ng/mL) against individual-predicted concentrations (IPRED, natural log scale, ng/mL); (C) DV against population-predicted concentrations (PRED, natural log scale, ng/mL); (D) Quantile–quantile (QQ) plot of the components of CWRES; (E) CWRES against PRED; (F) Relative frequency histogram of CWRES. Empty points in the graphs (A–E) represent each data value observed or estimated by the model.

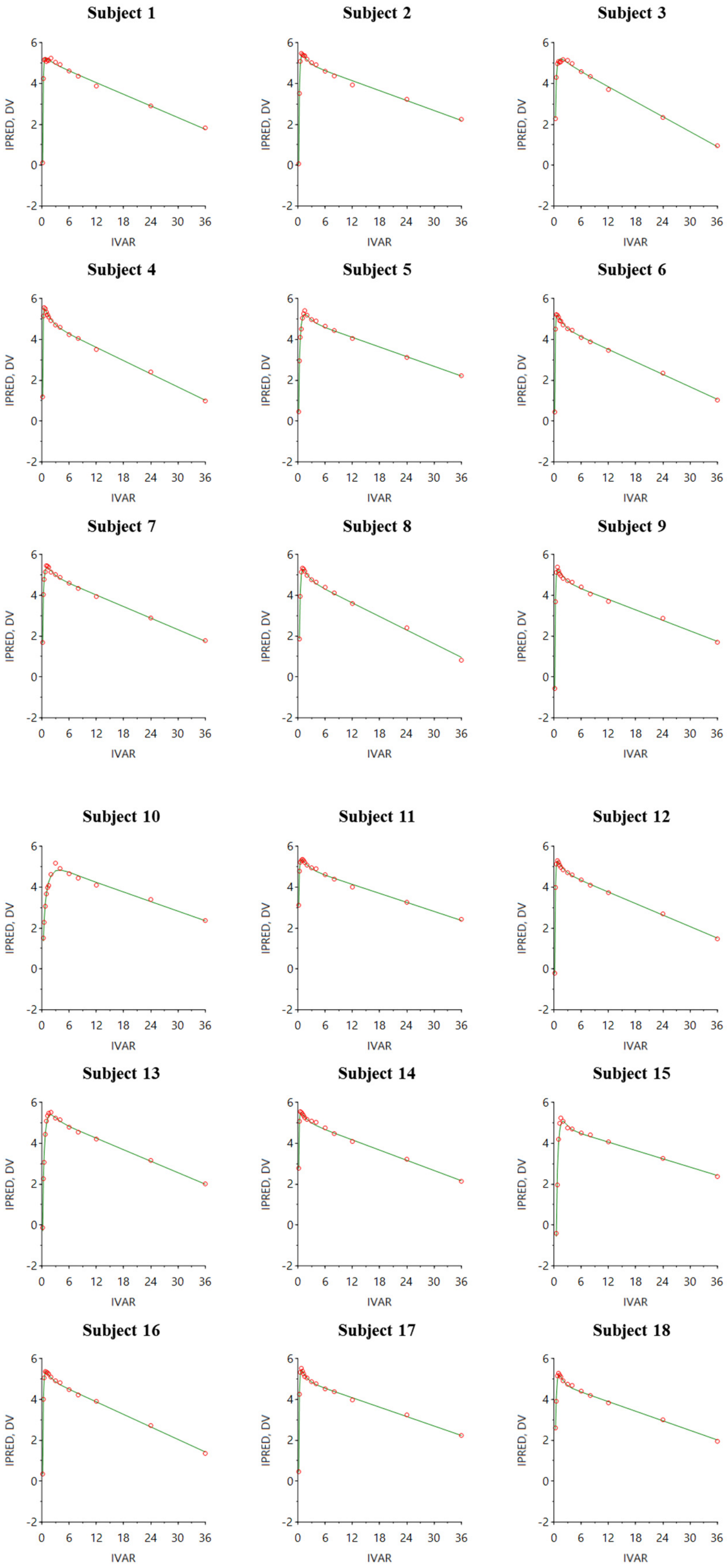
Subject 1
Subject 2
Subject 3
Subject 4
Subject 5
Subject 6
Subject 7
Subject 8
Subject 9
Subject 10
Subject 11
Subject 12
Subject 13
Subject 14
Subject 15
Subject 16
Subject 17
Subject 18
IPRED, DV
IVAR

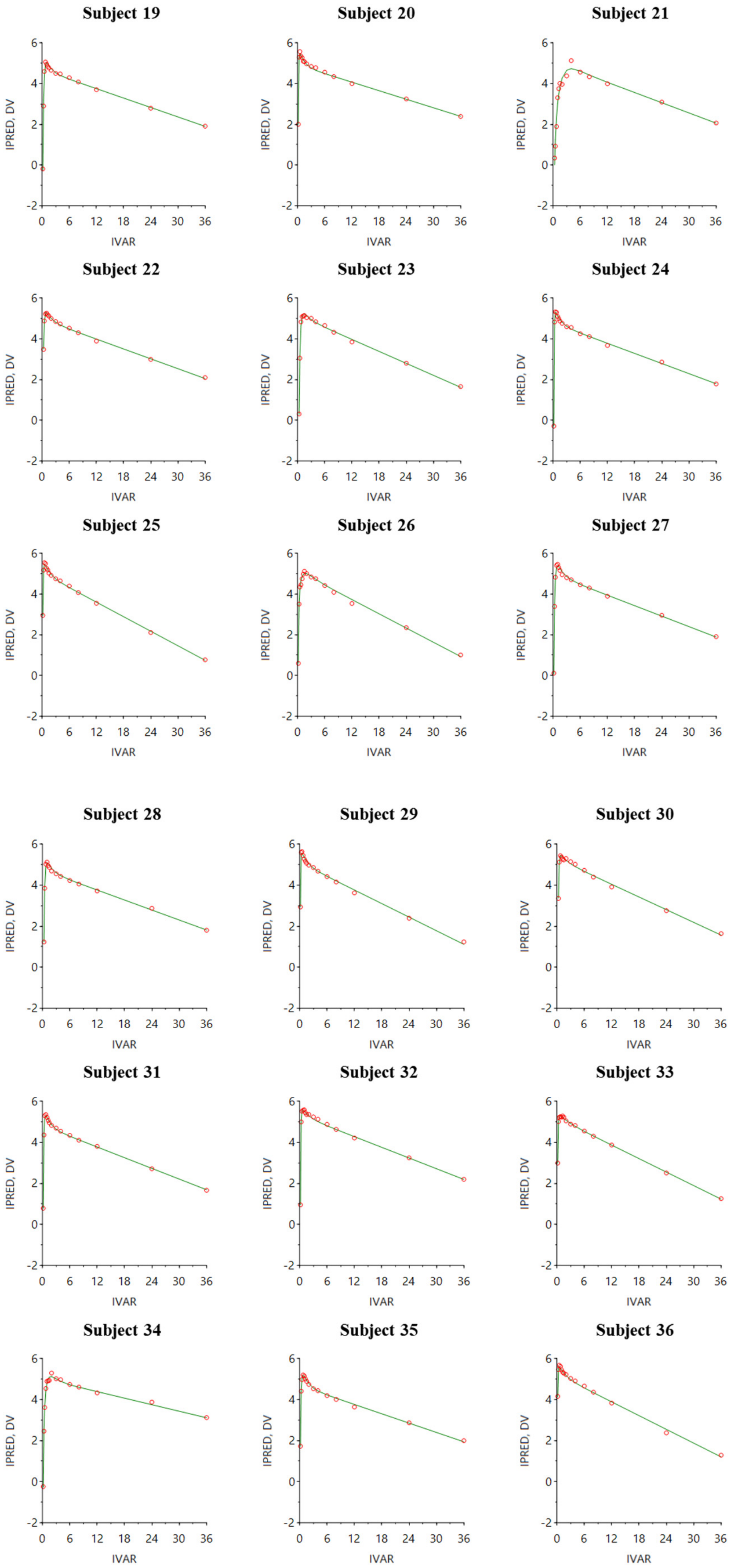
Subject 19
Subject 20
Subject 21
Subject 22
Subject 23
Subject 24
Subject 25
Subject 26
Subject 27
Subject 28
Subject 29
Subject 30
Subject 31
Subject 32
Subject 33
Subject 34
Subject 35
Subject 36
IPRED, DV
IVAR

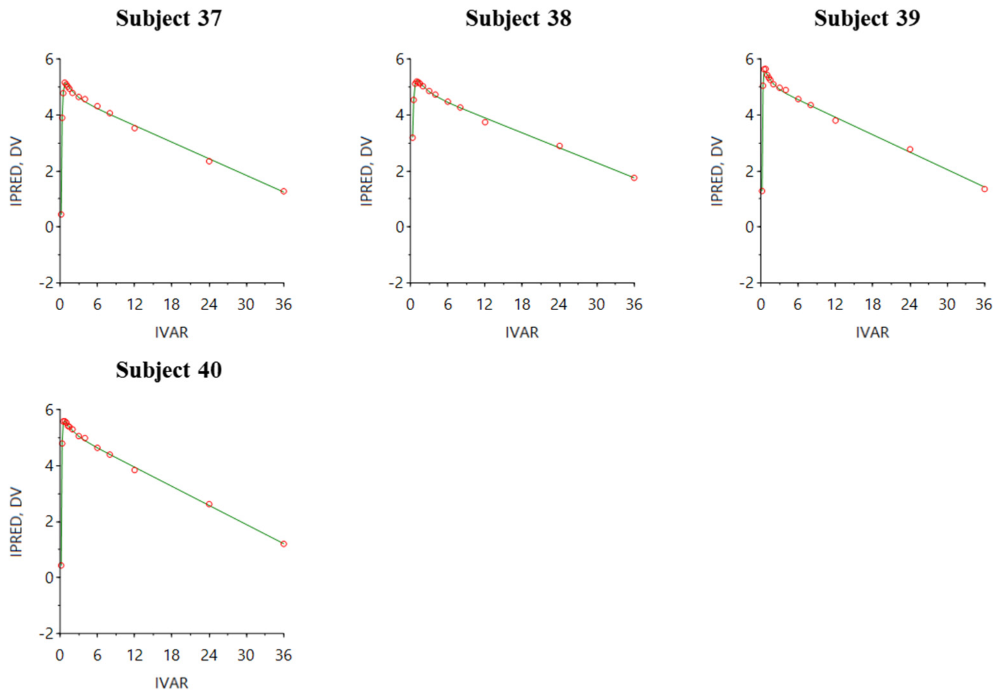

Figure S4. Subject-selective comparison of observed plasma concentrations (DV, natural log scale, ng/mL) over time (IVAR, h) after oral exposure to levocetirizine 5 mg tablets and individual-predicted concentrations (IPRED, natural log scale, ng/mL) estimated by the model. The empty dots and solid lines in the graphs represent observations and values predicted by the model, respectively.

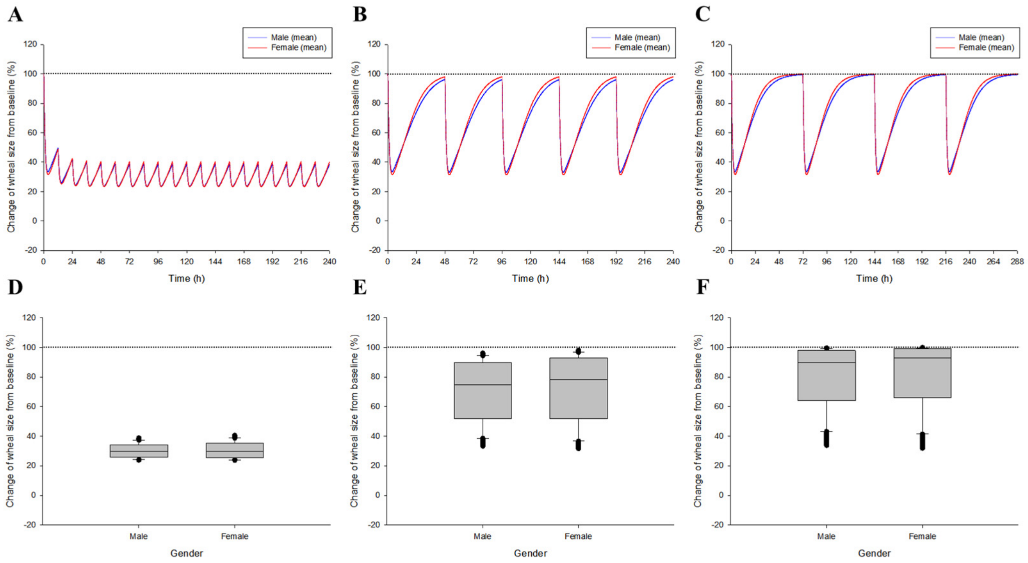

Figure S5. Model simulation results of wheal size change profiles following multiple oral exposures (5 mg tablet) at different dosing intervals (A, 12 h; B, 48 h; C, 72 h) to levocetirizine between genders after reflection of the covariates in the levocetirizine population pharmacokinetic model. A–C refer to the wheal size change profiles estimated by applying the mean biochemical parameter values for each gender to the covariate correlation within the model. D–F refer to the comparison of results between genders at steady state (A, 228–240 h; B, 192–240 h; C, 216–288 h) estimated by applying the mean biochemical parameter values in each gender to the covariate correlation within the model. The black dotted lines in the graph (A–F) represent the baseline (as no inhibition) of the histamine (H_1)-induced response.

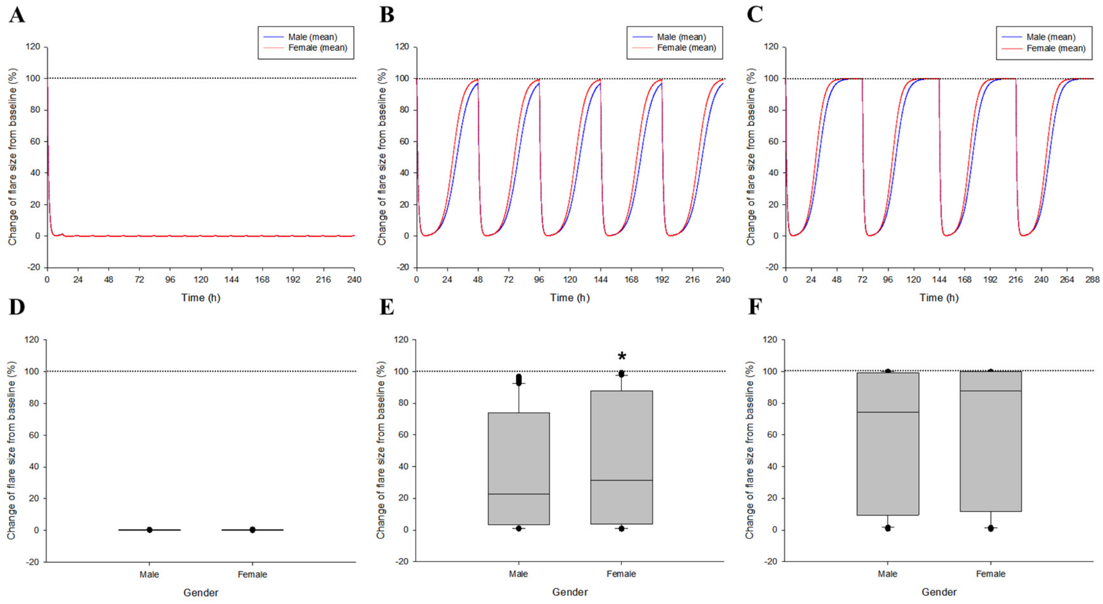

Figure S6. Model simulation results of flare size change profiles following multiple oral exposures (5 mg tablet) at different dosing intervals (A, 12 h; B, 48 h; C, 72 h) to levocetirizine between genders after reflection of the covariates in the levocetirizine population pharmacokinetic model. A–C refer to the flare size change profiles estimated by applying the mean biochemical parameter values for each gender to the covariate correlation within the model. D–F refers to the comparison of results between genders at steady state (A, 228–240 h; B, 192–240 h; C, 216–288 h) estimated by applying the mean biochemical parameter values in each gender to the covariate correlation within the model. The black dotted lines in the graph (A–F) represent the baseline (as no inhibition) of the histamine (H_1)-induced response. *$p < 0.05$ compared to the value in males.

Disclaimer/Publisher's Note: The statements, opinions and data contained in all publications are solely those of the individual author(s) and contributor(s) and not of MDPI and/or the editor(s). MDPI and/or the editor(s) disclaim responsibility for any injury to people or property resulting from any ideas, methods, instructions or products referred to in the content.
